# Pure Hydrogen and Methane Permeation in Carbon-Based Nanoporous Membranes: Adsorption Isotherms and Permeation Experiments

**DOI:** 10.3390/membranes14060123

**Published:** 2024-05-26

**Authors:** Matthis Kurth, Mudassar Javed, Thomas Schliermann, Georg Brösigke, Susanne Kämnitz, Suresh K. Bhatia, Jens-Uwe Repke

**Affiliations:** 1 DBFZ Deutsches Biomasseforschungszentrum Gemeinnützige GmbH, 116, 04347 Leipzig, Germany; 2 Dynamik und Betrieb Technischer Anlagen, Technische Universität Berlin, 10623 Berlin, Germany; 3 Fraunhofer Institut für Keramische Technologien und Systeme IKTS, 07629 Hermsdorf, Germany; 4 School of Chemical Engineering, University of Queensland, Brisbane 4072, Australia

**Keywords:** membrane, carbon, adsorption isotherms, Maxwell–Stefan surface diffusion

## Abstract

This paper presents the results of adsorption and permeation experiments of hydrogen and methane at elevated temperatures on a carbon-based nanoporous membrane material provided by Fraunhofer IKTS. The adsorption of pure components was measured between 90 °C and 120°C and pressures up to 45 bar. The Langmuir adsorption isotherm shows the best fit for all data points. Compared to available adsorption isotherms of H_2_ and CH_4_ on carbon, the adsorption on the investigated nanoporous carbon structures is significantly lower. Single-component permeation experiments were conducted on membranes at temperatures up to 220 °C. After combining the experimental results with a Maxwell–Stefan surface diffusion model, Maxwell–Stefan surface diffusion coefficients $D_{i}^{s}$ were calculated. The calculated values are in line with an empirical model and thus can be used in future multi-component modeling approaches in order to better analyze and design a membrane system. The published adsorption data fill a gap in the available adsorption data for CH_4_ and H_2_.

## 1. Introduction

### Motivation and Literature Review

Hydrogenation reactions are crucial for future renewable energy systems [1,2,3,4]. The efficient separation of synthesis gases downstream or in situ at process temperatures and pressures is a challenging task. This is usually achieved via adsorption processes [5]. However, either the in situ conditions are too severe for adsorption processes or these methods are not economically feasible if the plant is not large enough. This limits the applications of hydrogenation reactions for biorefinery applications [5,6,7,8].

Nanoporous carbon membranes are a promising option for gas separation with robust stability, e.g., for CO_2_ methanation reactions with temperatures up to 350 °C and pressures up to 50 bar, which is a possible biorefinery process [9,10,11]. Membrane applications in reactive systems have become increasingly important in past years [12,13]. Since Fraunhofer IKTS Hermsdorf develops nanoporous carbon membranes for various membrane reactor applications [13], such membranes were chosen for these experiments. These membranes may play an important role in the application of membrane reactors, and thus data on mass transport and adsorption are needed. The separation of CO_2_ methanation gas products mainly involves four components: H_2_, CH_4_, CO_2_ and H_2_O. Since the present research is the first to study this mass transport, only H_2_ and CH_4_ are considered in this analysis. Better understanding the gas separation principles of membranes and developing a mass transport model may provide us with an opportunity to optimize the performance of membrane systems. With modeling approaches, different process designs can be mathematically evaluated and compared.

Different membrane systems require different modeling approaches [3]. Dense membranes use the solution–diffusion approach, microporous membranes use viscous flow, and smaller pores use Knudsen diffusion. For nanoporous carbon membranes, the state-of-the-art approach is Maxwell–Stefan (MS) surface diffusion [14,15,16]. Krishna [14] developed a comprehensive approach using nanoporous media descriptions that uses adsorption isotherms to account for surface–molecule interactions. Monsalve et al. [15] used molecular simulations to provide data for research work, and Cardoso et al. [16] used multi-component adsorption isotherms based on literature data.

In order to use the Maxwell–Stefan approach, adsorption and permeation data are necessary. Looking at the publicly available data on H_2_ and CH_4_ adsorption on porous carbon material [17,18,19,20,21,22,23,24,25] and at the *Database of Novel and Emerging Adsorbent Materials* [26], only a small number of adsorption isotherms of gases on nanoporous carbon have been recorded for temperatures above 100 °C, which are the temperatures of interest for many industrial processes. For the measurement of the gas permeation of H_2_ and CH_4_ through a carbon membrane, the current literature also provides less data.

This publication aims to fill that gap. The pure component adsorption of H_2_ and CH_4_ is measured at temperatures up to 90 °C and pure component permeation is measured across a temperature range of 120 °C to 220 °C.

The pure component adsorption isotherm parameters and permeation data are then used to calculate Maxwell–Stefan surface diffusion coefficients $D_{i}^{s}$ across a temperature range of 120 °C to 220 °C for H_2_ and CH_4_ with the aid of a three-layered membrane model.

The main objective of this work is to provide permeation and adsorption data for H_2_ and CH_4_ on a nanoporous carbon membrane layer at elevated temperatures and to calculate Maxwell–Stefan surface diffusion coefficients. These are valuable data for further research and fill a gap in the literature. These parameters will be of value for different modeling approaches related to carbon membranes.

## 2. Experimental

The experiments were performed due to the described gap in the available literature data for nanoporous carbon materials. The adsorption experiments were performed on the cast nanoporous carbon material provided by a manufacturer. These membranes were used for the permeation experiments in order to obtain sufficient data to calculate Maxwell–Stefan surface diffusion coefficients.

**Figure 1:**
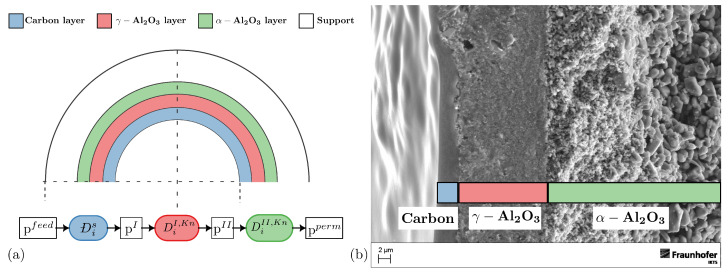
(**a**) The different layers of the membrane with their respective modeling approach. For the *carbon layer* with nano pores and for the two Al_2_O_3_ support layers, Maxwell–Stefan surface diffusion ($Di^{s}$) and Knudsen diffusion ($D_{i}^{Kn}$) are the principle of mass transport, respectively. The other layers above the two supporting layers are not considered in the model. (**b**) SEM picture of the membrane provided by *Fraunhofer IKTS*, Hermsdorf.

Single-component gases of H_2_ and CH_4_ were analyzed. The membranes and coating materials were prepared at Fraunhofer IKTS in Hermsdorf, Germany. A *Quantachrome Autosorb-iQ-C* analyzer at the DBFZ and a *RuboLab RuboSORB* magnetic suspension balance at TU Berlin, Germany, were used for the adsorption experiments. Table 1 presents the results of standard characterization techniques performed with the casting material in the laboratories of the DBFZ. Table 2 shows the values of the different layers provided by Fraunhofer IKTS. The layers that are shown are just a simplification of the more complex structure of the prepared membrane, which consists of more layers. These layers are not shown in Table 2 in order to not to infringe the intellectual property of Fraunhofer IKTS. Permeation experiments were performed on cylindrical membranes with a nanoporous carbon layer supported by two support layers (compare Table 2 and Figure 1). The membrane samples were 105 mm long and have an outer radius $r^{out}$ and an inner radius $r^{in}$ of 5 mm and 3.5 mm, respectively. An overview of all experiments and the respective results is presented in Table 3.

### 2.1. Membrane Preparation Procedure

Based on the publications [9,27], the membranes for the permeation experiments and the casting material for the adsorption experiments were prepared at Fraunhofer IKTS in Hermsdorf.

The required synthesis-grade chemicals, furfuryl alcohol (PFA) and pyrrole, were purchased from MERCK (Germany). Polyethylene glycol methyl ether 750 (PEG 750) and concentrated nitric acid (HNO_3_) were purchased from Sigma-Aldrich (St. Louis, MO, USA). The PFA precursor was synthesized according to an optimized formulation by Hucke [28]. The polymer composition consists of 30 mL of furfuryl alcohol, 6 mL of pyrrole, 15 mL of PEG 750 and 1 mL of concentrated nitric acid as a catalyst. The pyrrole was added to the furfuryl alcohol while stirring at room temperature. PEG 750 was melted and added to the mixture with rapid stirring. Concentrated nitric acid was then added slowly under cooling ( 0.05 mL/300 s) using a titrator (Titronic ®300, SI Analytics, Weilheim, Germany). After complete addition of the nitric acid, a black polymer solution was obtained. This polymer solution was used for membrane coatings using the same procedure for the provision of the casting material for the adsorption experiments.

Al_2_O_3_ single-channel tubes with a 5 nm α-Al_2_O_3_ membrane layer were used as supports. The openings of the single-channel tubes were closed with a glass seal. The inside of the tubes was coated by dip coating under clean room conditions. A semi-automatic coating system (developed by IKTS) was used to coat the inside of the tubes. The single-channel tube to be coated was immersed in the precursor solution up to the glass seal mark. A vacuum pump was applied to completely fill the tube with PFA solution. After a holding time of 60 s, the single-channel tube was evacuated with nitrogen at a flow rate of 60 mL/min. The PFA coatings were first dried for 24 h under clean room conditions and later cross-linked at a temperature of 80 °C for 4 h in air. Pyrolysis was applied to decompose the polymer coatings into carbon membranes. For this process, PFA-coated supports were heated to 500 °C under nitrogen (500 L h^−1^). At a pyrolysis temperature of 500 °C, the system was switched to argon (200 L h^−1^) and further heated to 670 °C. At a cooling temperature of 500 °C, the system was switched back to nitrogen.

The prepared membranes are cylindrical with an inner radius of 3.5 mm and an outer radius of 5 mm. A thin carbon membrane is supported by a series of α-Al_2_O_3_ and γ-Al_2_O_3_ ceramics with different pore sizes. The different layers of the membrane are shown in Table 2 with their respective pore sizes $d_{p}$, layer thicknesses δ, main material, porosity ε and calculated Knudsen number at 120 °C. A representation of the membrane is visualized in Figure 1. The manufactured membrane consists of more than the layers shown in Figure 1, but only the first three layers are shown and included in the modeling of mass transport. The reason for this is that the detailed production process used by the manufacturer Fraunhofer IKTS cannot be revealed. Secondly, based on an analysis of the individual mass transport resistances, the assumption was made that only two support layers should be considered. All other layers are considered irrelevant. The first carbon layer with surface diffusion and the two α-Al_2_O_3_ and γ-Al_2_O_3_ layers with Knudsen diffusion (see Figure 1) are considered to affect mass transport and are therefore implemented in the model.

All subsequent layers are treated as a single support layer (see Figure 1), which affects the mass transport.

### 2.2. Adsorption Measurements

As shown in Figure 1, the carbon layer is modeled using Maxwell–Stefan surface diffusion, where an understanding of the adsorption is necessary to calculate the mass transport. The two support layers are modeled using Knudsen diffusion, where adsorption is not considered in the mass transport description as described by [29,30].

#### 2.2.1. Casting Material Characterization Measurements

In order to better understand the different adsorption results, several tests were performed on the cast material prior to the adsorption analysis. The textural properties of the membranes were characterized by gas sorption using a *3P micro 200 Porosity Analyzer (3P Instruments)* and an *Autosorb-iQ-C (Quantachrome)* at the laboratory at the DBFZ in Leipzig. Different adsorptives were used to characterize pores ranging from sub-nanometer scales to several hundred nanometers. Measurements with nitrogen at 77 K were performed on a *Micro 200* using liquid nitrogen. An *Autosorb-iQ-C* equipped with a *CryoTune 195 (3P Instruments)* was used for CO_2_ sorption at 273 K. Prior to analysis, the membranes were pretreated under a vacuum for 30 min at 90 °C and 12 h at 250 °C to desorb pre-adsorbed molecules. A minimum sample mass of 250 mg was used.

At the DBFZ, several characterization experiments were conducted on the casting material. Nitrogen sorption at 77 K was studied to determine the specific surface area ($S_{BET}$) according to Brunauer, Emmett and Teller (BET) [31] theory and the micropore volume ($V_{mic}$) according to Dubinin–Radushkevich (DR) [31] theory. The total pore volume ($V_{T}$) was determined at a relative pressure of $p_{rel}$ 0.995. CO_2_ sorption data were evaluated using the Dubinin–Radushkevich (DR) method as well as Monte Carlo and NLDFT models of carbon. More details on Monte Carlo and DFT methods can be found in [32]. All characterization results are presented in Table 1. As can be seen from the range of values for the parameters, the measurement of pore sizes in these ranges is difficult. The characterization based on N_2_ BET is obviously not suitable for the small pores used. The value of the apparent pore size of 0.4 nm is taken from the documentation provided by Fraunhofer IKTS and the values in Table 1 were measured for a better understanding of the material.

#### 2.2.2. Recording Adsorption Isotherms

Based on the characterization measurements, adsorption isotherm experiments for H_2_ and CH_4_ on the casting material of the carbon membrane were carried out at TU Berlin. The characteristics of the adsorbent are shown in Table 1, which were determined using standard methods such as N_2_ and CO_2_ adsorption at the DBFZ. Before the measurement, the sample was pretreated under a vacuum at 150 °C for 2 h to desorb any moisture content. For the pure gases of CH_4_ and H_2_, the adsorption was measured at 90 °C, 110 °C and 120 °C with the *RuboSORP* magnetic suspension balance. The pressure of the pure gas for each experimental trial was initiated at 5 bar and then increased to 45 bar with pressure increments of 5 bar. The pressure was maintained until an equilibrium was reached with a maximum of 4 h and then maintained for 2 h for the measurement to be taken.

#### 2.2.3. Assumptions and Experimental Errors

The detectable amount of adsorbate decreases at higher temperatures. Therefore, the maximum temperature at which adsorption properties can be measured is limited by the sensitivity of the balance. In the case of the *RuboSORB* magnetic suspension balance, the maximum temperature for gas adsorption for the tested material is 120 °C due to limitations in the measuring range. H_2_O residues of 20 ppm for H_2_ and 100 ppm for CH_4_ were detected in the gas containers used. However, these are not expected to have a significant effect on the conducted tests.

The experimental error of the *RuboSORB* is given as 0.01 mg. The resulting error in the adsorption isotherm was calculated using *Monte Carlo* simulations [33,34] using the residuals of the curves with the estimated parameters and the measured values as the error of the parameter estimation [35]. The parameters of different adsorption isotherms were estimated using the non-linear optimization *CasADi* software package [36] with the *Ipopt* solver, implemented in the *mopeds* framework.

### 2.3. Permeation Experiments

Single gases of H_2_ and CH_4_ were applied to the feed side of the membrane at different pressures and temperatures and the permeation behavior was characterized based on the given permeate pressure and the resulting permeate flux $F_{i}^{perm}$, where the subscript _*i*_ indicates the species. Using the membrane characteristics, the permeation can be calculated (compare Equation (1)), where $A^{mem}$ and $\Delta p_{i}$ are the membrane surface in m^2^ and the pressure drop in bar.

(1) $$\begin{array}{cc} P_{i} & =\frac{F_{i}^{perm}}{A^{mem}\Delta p_{i}} \end{array}$$

#### 2.3.1. Experimental Setup and Method

**Figure 2:**
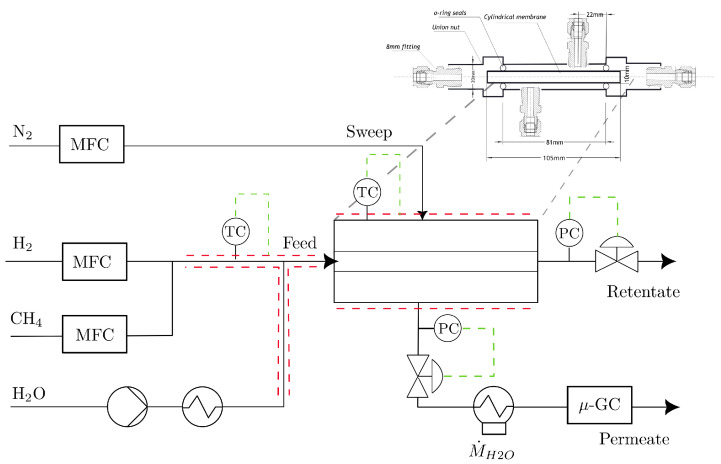
Simplified A PID of the used permeation testing rig with a gas inlet on the left-hand side and the permeate stream analysis system on the right-hand side. Pipe trace heaters and heating jackets were used to set the necessary temperatures are shown in red. The control loops are shown in green.

The membranes are cylindrical and have a length of 105 mm with an inner radius of 3.5 mm and an outer radius of 5 mm. The permeation experiments were performed using a permeation test rig (the simplified PID shown in Figure 2), where the membrane was mounted inside a testing cell with O-rings. The temperature was varied across a temperature range between 120 °C and 220 °C. The pressure was varied in different steps between 2 bar and 15 bar. The gas composition of the permeate stream was analyzed using an *Inficon μ-GC* downstream of the permeation test rig.

The permeation side of the membrane was flushed with a sweep stream of 50 L h^−1^ (STP) of N_2_, and before each experimental point, the system was held at the experimental temperature for 2 h.

The dry gas composition was analyzed using a *Micro GC Fusion* system from *inficon*.

Knowing the sweep flow ($F^{Sweep}$) in mol min^−1^ and the composition ($x_{i}$) of the permeate stream (by the gas chromatic analysis) and assuming that the ideal gas law is valid, the permeation flow $F_{i}^{perm}$ through the membrane can be calculated with Equation (2).

(2) $$\begin{array}{cc} F_{i}^{perm} & =\frac{x_{i}\;\cdot \;F^{sweep}}{x_{N_{2}}} \end{array}$$

The temperature, permeate pressure ($p^{perm}$) and retentate pressure ($p^{ret}$) were varied according to a statistical design of experiments (see Supporting Material). The temperature was varied between 120 °C and 220 °C. For CH_4_, the feed pressure was varied between 5 bar and 15 bar. For H_2_, the pressure was varied between 2 bar and 5 bar. In order to minimize the systematic error, a *design of experiments* with a factorial/RSM design was set up and the set points for the pressure and temperature levels were changed accordingly. Each experimental point was measured over 30 min.

#### 2.3.2. Limitations, Assumptions, and Experimental Error

The pressure ranges of CH_4_ and H_2_ could not be the same because H_2_ permeates very easily through the membrane such that the mass flow controller could not provide a mass flow to set a feed pressure higher than 5 bar. The error of the permeation measurements was calculated by the standard deviation of the readings. The system was assumed to be in equilibrium for the duration of the experiment.

### 2.4. Diffusion Coefficient Calculation

The membrane consists of three layers, as shown in Figure 1. Based on the adsorption and the permeation data, a single-component mass transport model was developed to calculate the Maxwell–Stefan and Knudsen diffusion coefficients in the carbon and in the support layers of the membrane, respectively (see Figure 1 and Equations (10) and (8)).

The thickness of the effective carbon membrane layer (<2 μm) is negligibly small compared to the inner radius of the membrane, which is 3.5 mm. The two supporting layers do not impose a high resistance towards mass transport. To simplify the modeling approach, the membrane was treated as a flat surface and Cartesian coordinates were used.

#### 2.4.1. Adsorption Isotherm Temperature Extrapolation

The adsorption experiments were performed at temperatures of between 90 °C and 120 °C and the permeation experiments were performed at temperatures ranging from 120 °C to 220 °C. Therefore, the adsorption isotherms must be extrapolated in order to be used to calculate the diffusion coefficients. There are several approaches to extrapolating adsorption isotherms, including the Langmuir and Dubin–Radushkevish isotherms [16,37].

In the Langmuir isotherm, the isosteric heat of adsorption (compare Equation (3)) is used to extrapolate the parameters $q_{i,sat}$ and $K_{i}$ (cf. Equations (6) and (7)) by estimating the parameter *X* and calculating the isosteric heat of adsorption $Q_{i,st}$ (see Equation (4)) and thus the amount adsorbed at another temperature.

(3) $$\begin{array}{cc} Q_{i,st} & =RT^{2}{\left(\frac{\partial \ln p_{i}}{\partial T}\right)}_{n_{i}} \end{array}$$

(4) $$\begin{array}{cc} Q_{i,st} & =E_{i} \end{array}$$

(5) $$\begin{array}{cc} K_{i} & =K_{i}^{0}\exp\left(\frac{E_{i}}{RT}\right) \end{array}$$

(6) $$\begin{array}{cc} q_{sat} & =q_{sat,0}\exp\left[X\left(1-\frac{T}{T_{0}}\right)\right] \end{array}$$

(7) $$\begin{array}{cc} K_{i} & =K_{i,0}\exp\left[\frac{Q_{i,st}}{RT}\left(\frac{T_{0}}{T}-1\right)\right] \end{array}$$

This extrapolation introduces an additional error into the calculation at other temperatures. Therefore, this error was estimated via a Monte Carlo simulation and can be used in the further calculation of the diffusion coefficients. The application of the Monte Carlo simulation is described in detail in the Supplementary Materials.

#### 2.4.2. Description of the Different Layers

The given membrane consists of three different layers with different pore sizes and thicknesses, as shown in Table 2 and Figure 1.

Starting from the Maxwell–Stefan surface diffusion description for one component, the integration over the length of the membrane leads to the formulation in Equation (8).

(8) $$\begin{array}{cc} D_{i}^{s} & =-\frac{N_{i}^{s}p_{i}}{\rho \varepsilon \theta_{i}} \end{array}$$

The Knudsen numbers of the layers are in the range 0.1–10 (see Table 2) for the first two Al_2_O_3_ layers, representing a transition region where fluid–fluid interactions, which can be described by viscous flow, may contribute to the flow. As a simplification, this influence is neglected based on a comparison between Knudsen and viscous flow. The Knudsen number for the support layer is outside the Knudsen regime and only a small resistance to mass transport is attributed to these layers. Thus, the α Al_2_O_3_ layers with a pore diameter greater than $d_{p}≧100\;nm$ are neglected in the description of mass transport through this membrane.

For the porous carbon layer with a pore size of about $d_{p}=0.42\;nm$ [27], Knudsen flow is not applicable because the molecules inside these narrow pores are always under the influence of the pore wall’s force field [38].

As described earlier, the transport of gases through mesoporous media can be attributed to different transport mechanisms; one simple approach is Knudsen diffusion, as described in [29,30], where the approach is compared with other approaches for the Al_2_O_3_ layer. For the support layer of α-Al_2_O_3_ with an average pore size of $d_{p}\approx$ 70 nm, Gao et al. have shown that Knudsen diffusion is dominant, with at least 80 % of the mass transport resistance [29]. Viscous flow is shown to be insignificant in this region based on a comparison of mass transport resistances. For the interlayer of γ-Al_2_O_3_ with a mean pore size of about $d_{p}\approx$ 5 nm, Knudsen diffusion is applicable, but it overestimates the diffusion of gases. Gao et al. suggest an alternative approach for this region, namely the *oscillator model*, but acknowledge that Knudsen diffusion is a useful approach [30]. Consequently, Knudsen diffusion is used for the two support layers.

As an example, the Knudsen diffusion flow for the *α*-Al_2_O_3_ layer can be defined as in [39]:

(9) $$\begin{array}{cc} N_{i} & =-D_{i}^{II,Kn}\frac{p_{i}^{II}-p_{i}^{perm}}{RT\Delta \delta^{\alpha }} \end{array}$$

where $\delta^{\alpha }$ and $p_{i}^{II}$ are the thickness of the intermediate layer and the pseudo partial pressure at the interface between the γ and the α interlayer. This description is used for both layers.

Following the previously defined descriptions for individual gas transport through different porous layers (Equations (10)–(12)) by Knudsen diffusion, the partial pressure difference between each layer can be calculated (see Figure 1). Thus, knowing the measured flow (Equation (13)), the MS diffusion coefficients $D_{i}^{s}$ can be calculated (Equation (14)).

Carbon layer:
  (10) $$N_{i}^{s}\int_{0}^{\delta_{C}}\;dz=-D_{i}^{s}q_{i}^{sat}\varepsilon^{C}\rho^{C}\int_{p_{i}^{feed}}^{p_{i}^{II}}\;\frac{\theta_{i}}{p_{i}}dp_{i}$$
γ-Al_2_O_3_ layer:
  (11) $$N_{i}^{Kn,\gamma }=\frac{4}{3}\frac{\varepsilon^{\gamma }}{\tau^{\gamma }}\frac{d_{p}^{\gamma }}{\sqrt{2\pi RTM_{i}}}\frac{p_{i}^{II}-p_{i}^{I}}{\delta^{\gamma }}$$
α-Al_2_O_3_ layer:
  (12) $$N_{i}^{Kn,\alpha }=\frac{4}{3}\frac{\varepsilon^{\alpha }}{\tau^{\alpha }}\frac{d_{p}^{\alpha }}{\sqrt{2\pi RTM_{i}}}\frac{p_{i}^{II}-p_{i}^{perm}}{\delta^{\alpha }}$$
And with:
  (13) $$N_{i}^{Kn,\alpha }=N_{i}^{Kn,\gamma }=N_{i}^{s}$$
one can calculate:
  (14) $$D_{i}^{s}=-\frac{N_{i}^{s}\delta_{C}}{q_{i}^{sat}\epsilon^{C}\rho^{C}\int_{p_{i}^{feed}}^{p_{i}^{II}}\;\frac{\theta_{i}}{p_{i}}dp_{i}}$$

In this study, a series of adsorption and permeation experiments were carried out on a membrane and a cast material of the same type to provide pure component data for the development of a three-layer Maxwell–Stefan surface diffusion model. Using the developed model and the permeation experiments, the Maxwell–Stefan surface diffusion coefficients can be calculated for temperatures between 120 °C and 220 °C. The results are presented in the following section.

## 3. Results

The aim of this study is to provide adsorption isotherms for the components (Section 3.1), to present experimental results of the permeation of pure H_2_ and CH_4_ through a membrane of the same material and finally to present the calculated Maxwell–Stefan surface diffusion coefficients of the pure components (Section 3.3) based on a multilayer model.

The adsorption was measured on a *RuboLab* magnetic suspension balance, the permeation was measured in a permeation test rig and the Maxwell–Stefan surface diffusion coefficients were calculated using a multi-layer model of the membrane, shown in Figure 1. An overview of all experiments and results is presented in Table 3.

### 3.1. Adsorption Measurements

**Figure 3:**
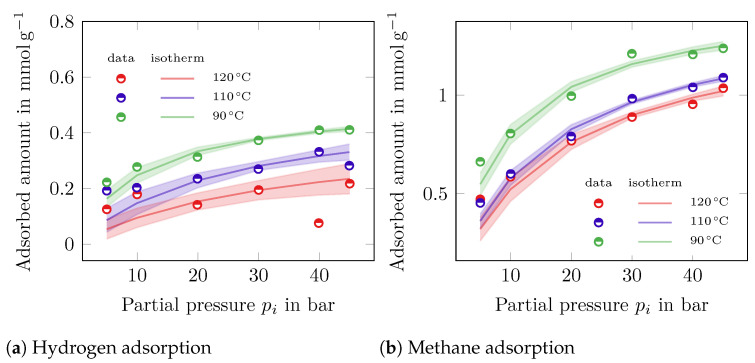
Adsorbed amount of H_2_ (**a**) and CH_4_ (**b**) at 120 °C (red), 110 °C (blue) and 90 °C (green). The calculated *Langmuir* isotherm is shown by the solid line. The calculated Monte Carlo simulation deviation is shown in transparent colors.

The adsorbed amount of CH_4_ and H_2_ over the partial pressure on the carbon casting material is shown in Figure 3. For both components, the *Langmuir* isotherm is shown with the respected error range based on a Monte Carlo simulation described in the Supplementary Materials.

The estimated coefficients are presented in Table 4, where the coefficients for the *Langmuir*, *Freundlich*, and the *Toth* isotherms are marked with the respective superscripts (^*l*^, ^*fr*^, and ^*t*^). The coefficient of determination ($R^{2}$) is shown for each parameter estimate. The *Langmuir* and *Freundlich* isotherms lead to a good parameter estimation for most data points. However, for H_2_ at 120 °C, the error is considerably higher compared to the other values due to the low amount of adsorbed material on the surface. Although the coefficient of determination of the *Toth* isotherm looks promising at first glance, the parameters do not show a physically sensible range, since the value for the saturation $q_{i,sat}^{t}$ is calculated to be at the very upper limit of the parameter estimation used. It could be concluded that six data points are not sufficient to estimate three parameters with such a high standard deviation. Therefore, it is not recommended to use the *Toth* isotherm in this context.

The isosteric heat of adsorption $Q_{st}$ for both components is calculated, using the *Langmuir* isotherm, to be $Q_{st}^{H_{2}}=11.03\;kJ\;\mathrm{mol}$^−1^ and $Q_{st}^{{CH}_{4}}=27.44\;kJ\;\mathrm{mol}$^−1^.

### 3.2. Permeation Experiments

**Figure 4:**
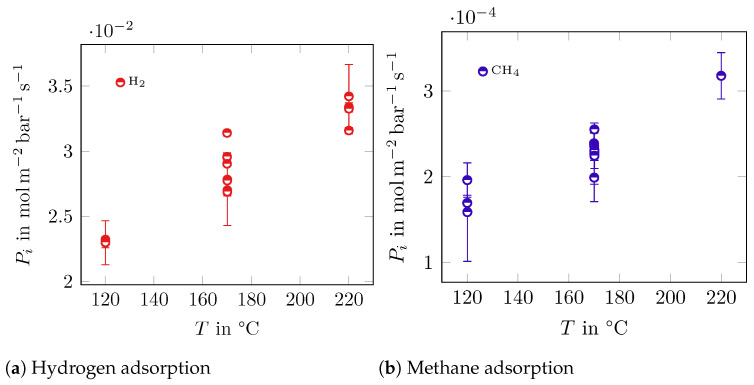
Single-component permeance $P_{i}$ in mol m^−2^ bar^−1^ s^−1^ over the temperature in °C of H_2_ and CH_4_.

In Figure 4, the single-component permeance versus temperature is shown. H_2_ shows a permeance of about 1 × 10^−2^ mol m^−2^ bar^−1^ s^−1^, which is consistent with other tested carbon molecular sieve membranes in [9,40]. CH_4_ shows permeances with values of 2 × 10^−4^ mol m^−2^ bar^−1^ s^−1^. As the temperature increases, so does the permeance for both components.

### 3.3. Calculation of MS Diffusion Coefficients of Single Components

The Maxwell–Stefan diffusion coefficients $D_{i}^{s}$ were calculated using the three-layer model by using the adsorption data at temperatures up to 120 °C, temperature extrapolation, and permeation experiments at temperatures between 120 °C and 220 °C. The MS surface diffusion coefficients for H_2_ and CH_4_ are listed in Table 5 for the different temperatures analyzed.

The diffusion coefficient $D_{i}^{s}$ of H_2_ ranges from 9.81 × 10^−6^ m^2^ s^−1^ at 120 °C to 2.01 × 10^−5^ m^2^ s^−1^ at 220 °C. The diffusion coefficients $D_{i}^{s}$ of CH_4_ range from 3.73 × 10^−9^ m^2^ s^−1^ at 120 °C to 6.39 × 10^−9^ m^2^ s^−1^ at 220 °C. The Maxwell–Stefan surface diffusion coefficients of both components increase with the system temperature.

**Figure 5:**
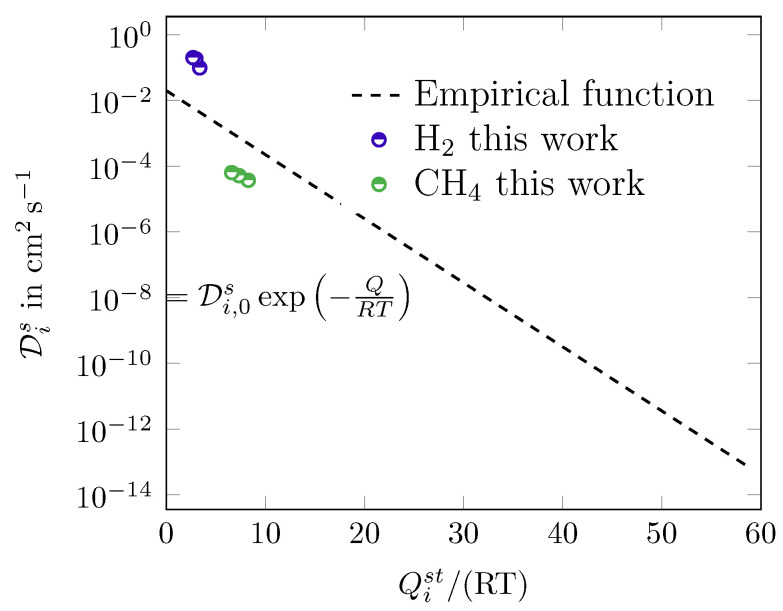
$D_{i}^{s}$ over $Q_{i}^{st}/\left(RT\right)$. The empirical function is based on [1] and [41].

In [1], an empirical correlation for the temperature dependency of Maxwell–Stefan surface diffusion coefficients is provided. In Figure 5, the calculated values are plotted against the empirical function. The values of the results are comparable to the ones from the empirical function and thus comparable to those from the literature measured at lower temperatures. Since the objective of this work was to calculate values for $D_{i}$ for an elevated temperature range, this can be taken as an validation of the values of the Maxwell–Stefan surface diffusion coefficients.

## 4. Discussion

**Figure 6:**
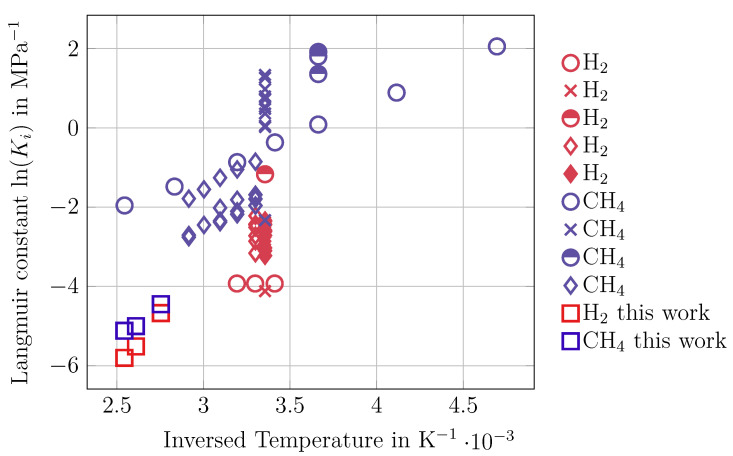
Comparison of the available equilibrium constants from adsorption of hydrogen ([18,22,23,24,25]) and methane [19,20,21,22] on different activated carbon materials at different temperatures available in [26] with the published data of this work. Note that the carbon materials for each source cited are not the same and thus measured at one distinct temperature with different adsorptive behavior.

The adsorption and permeation experiments performed provided the needed data to calculate Maxwell–Stefan surface diffusion coefficients. In this section, the provided results are compared to available publications. One important parameter is the equilibrium constant $K_{i}$ of the adsorption isotherm, which can be calculated from the measured adsorption isotherms using the *Langmuir* isotherm. These values can be compared with those found in the *NIST* database [26]. These were obtained for activated carbons with larger pore sizes. The comparison shown in Figure 6 can therefore be interpreted as follows.

The adsorption constants of CH_4_ and H_2_ are smaller compared to the literature data. Due to the small pore size of nanoporous carbon of 0.4 nm, the relatively large CH_4_ molecule (kinetic diameter of 0.38 nm) is expected to permeate slowly, and due to the inhibited adsorption, small H_2_ molecules with a kinetic diameter of 0.289 nm are expected to permeate faster. The low saturation capacities ($q_{i,sat}$) may be due to the small micropore volume.

Figure 6 also shows that data for this temperature range are still scarce and the identified error requires additional experiments.

The values for $Q_{i,st}$ are significantly higher than the published data [42,43]. For CH_4_, the range is usually between 16 kJ mol^−1^ and 19 kJ mol^−1^. In [43], $Q_{i,st}$ is given as 23 kJ mol^−1^. For H_2_, the range is usually between 6 kJ mol^−1^ and 8 kJ mol^−1^ [42]. The measured data differ from the theoretical predictions. This could be due to uncertainties in the measurement of the adsorption isotherms. Another factor is the pore structure of the material with pore widths of 0.4 nm, which may somewhat limit the full availability of all adsorption sites for molecules. While exact calculation methods were not feasible due to the high temperature, simplified approaches provided a valuable estimate with a tolerable error margin like described in Equation (5). Considering the high temperature and taking into account the high margin of error, the values for 120 °C remain well within the range suitable for further application.

The permeance values in the individual gas tests are in a range comparable to other publications using membranes with comparable pore diameters such as [27,44]. The permeance of H_2_ and CH_4_ increases with the temperature, as shown in Figure 4, which reflects what is shown in Table 5, where the Maxwell–Stefan surface diffusion coefficients $D_{i}$ increase with temperature as well.

Due to the performed temperature extrapolation (compare Equation (6) and Equation (7)), there is a margin of error in the adsorption isotherm which is diffucult to quantify. The values of $D_{i}$ and the development of the values with temperature are physically sensible.

## 5. Conclusions

The aim of this study was to provide adsorption and permeation data at elevated temperatures for H_2_ and CH_4_ and calculate the Maxwell–Stefan surface diffusion coefficients.

The derived parameters for the *Langmuir* and *Freundlich* isotherms provide valuable insights for further research. In addition, the permeation experiments performed, together with published data, provide a comprehensive data set for a comparative analysis of existing membranes.

All these objectives were achieved with a certain margin of error that needs to be considered when using the results. The Maxwell–Stefan surface diffusion coefficients ($D_{i}$) obtained from this study, combined with the experimental results, provide a strong basis for the development of a multi-component model. This model can be further validated and compared with existing binary permeation data. Future investigations will include performing binary permeation experiments on the same membrane under similar temperature conditions and using the data generated to refine the multi-component modeling approach.

## Figures and Tables

**Table 1:** Properties of the cast membrane material used for adsorption experiments and the conducted characterization method.

S_*mic*_ m^2^g^−1^	V_*mic*_ cm^3^g^−1^	d_*p*_ nm	V_*T*_ cm^3^g^−1^	(Estimation Method)
373	0.134	0.63	-	CO_2_ at 273 K (Monte Carlo)
385	0.127	0.57	-	CO_2_ at 273 K (NLDFT)
415	0.156	1.03	-	CO_2_ at 273 K (DR)
6	<0.001	-	<0.01	N_2_ at 77 K (BET & DR)

**Table 2:** The different layers of the membranes produced by Fraunhofer IKTS and the corresponding Knudsen number of H_2_ at 120 °C in these layers.

Layer	d_*p*_ m	*δ* μm	Material	*ε* %	*Kn* of H_2_ at 120 °C	Mass Transport Mechanism
Support	3.00 × 10^−6^	1500	α-Al_2_O_3_	32–35	4.87 × 10^−3^	None
Coarse UF	7.00 × 10^−8^	150	α-Al_2_O_3_	45	2.09 × 10^−3^	Knudsen
Fine UF	5.00 × 10^−9^	10	γ-Al_2_O_3_	55	2.92	Knudsen
Porous carbon	4.00 × 10^−10^	2	Carbon	45	3.48 × 10^1^	MS surface diffusion

**Table 3:** Overview of the different experiments, the conditions and the resulting data.

Experiment	Temperature K	Pressure bar	Material	Result
H_2_ adsorption	363/383/393	5–45	casting material	adsorption isotherm
CH_4_ adsorption	363/383/393	5–45	casting material	adsorption isotherm
CO_2_ adsorption	273	1	casting material	characterization
N_2_ adsorption	77	1	casting material	characterization
H_2_ permeation	393/443/493	2–5	cylindrical membrane	permeation data
CH_4_ permeation	393/443/493	5–15	cylindrical membrane	permeation data

**Table 4:** Estimated parameters of the *Langmuir* (superscript ^*l*^), *Freundlich* (superscript ^*fr*^) and *Toth* (superscript ^*t*^ isotherms for H_2_ and CH_4_ at temperatures of 90 °C, 110 °C and 120 °C.

Component	T °C	$q_{i,\mathrm{sat}}^{l}$ mmol g^−1^	$K_{i}^{l}$ bar^−1^	*R* ^2^	$K_{i}^{\mathrm{fr}}$	$n_{i}^{\mathrm{fr}}$	*R* ^2^	$K_{i}^{t}$ bar^−1^	$q_{i,\mathrm{sat}}^{t}$ mmol g^−1^	$f_{i}^{t}$	*R* ^2^
H_2_	90	0.093	0.512	0.980	0.132	0.302	0.993	1.000	0.262	1.377	0.991
H_2_	110	0.040	0.513	0.915	0.069	0.409	0.920	0.126	0.269	2.000	0.919
H_2_	120	0.030	0.407	0.147	0.007	1.000	0.138	0.030	1.000	0.407	0.147
CH_4_	90	0.117	1.486	0.984	0.413	0.296	0.985	0.117	1.000	1.486	0.984
CH_4_	110	0.067	1.442	0.993	0.253	0.386	0.997	0.096	0.605	1.923	0.996
CH_4_	120	0.060	1.399	0.993	0.253	0.366	0.998	0.108	0.531	2.000	0.998

**Table 5:** Calculated Maxwell–Stefan diffusion coefficients of H_2_ and CH_4_ at 120 °C, 170 °C and 220 °C in m^2^ s^−1^. The error range shown represents the standard deviation of the calculated values with the Monte Carlo simulation.

Component	120 °C m^2^s^−1^	170 °C m^2^s^−1^	220 °C m^2^s^−1^
H_2_	9.81 × $10^{-6}$ ± 2.55 × $10^{-6}$	1.84 × $10^{-5}$ ± 3.34 × $10^{-6}$	2.01 × $10^{-5}$ ± 4.92 × $10^{-6}$
CH_4_	3.73 × $10^{-9}$ ± 6.28 × $10^{-10}$	5.17 × $10^{-9}$ ± 3.54 × $10^{-10}$	6.39 × $10^{-9}$ ± 6.07 × $10^{-10}$

## Data Availability

The original contributions presented in the study are included in the article and Supplementary Materials, further inquiries can be directed to the corresponding authors.

## Supplementary Materials

The following supporting information can be downloaded at: https://www.mdpi.com/article/10.3390/membranes14060123/s1, Document S1: permeation data and adsorption data.

## Abbreviations

The following abbreviations are used in this manuscript:

PID	Piping and instrumentation diagram
MS	Maxwell–Stefan

## References

[1] Yang R.T., Yang R.T. (1987). CHAPTER 4—Rate Processes in Adsorbers. Gas Separation by Adsorption Processes.

[2] Gugliuzza A., Basile A. (2014). Membranes for Clean and Renewable Power Applications.

[3] Melin T., Rautenbach R. (2007). Membranverfahren: Grundlagen Der Modul- Und Anlagenauslegung.

[4] Rohde M.P. (2010). In-Situ H_2_O Removal via Hydorphilic Membranes during Fischer-Tropsch and Other Fuel-Related Synthesis Reactions. Ph.D. Thesis.

[5] Kohl A., Nielsen R. (1997). Gas Purification.

[6] Catarina Faria A., Miguel C.V., Rodrigues A.E., Madeira L.M. (2020). Modeling and Simulation of a Steam-Selective Membrane Reactor for Enhanced CO_2_ Methanation. Ind. Eng. Chem. Res..

[7] Ohya H., Fun J., Kawamura H., Itoh K., Ohashi H., Aihara M., Tanisho S., Negishi Y. (1997). Methanation of Carbon Dioxide by Using Membrane Reactor Integrated with Water Vapor Permselective Membrane and Its Analysis. J. Membr. Sci..

[8] Kaltschmitt M. (2019). Energy from Organic Materials (Biomass): A Volume in the Encyclopedia of Sustainability Science and Technology.

[9] Weyd M., Richter H., Puhlfürß P., Voigt I., Hamel C., Seidel-Morgenstern A. (2008). Transport of Binary Water–Ethanol Mixtures through a Multilayer Hydrophobic Zeolite Membrane. J. Membr. Sci..

[10] Simon A., Seyring M., Kämnitz S., Richter H., Voigt I., Rettenmayr M., Ritter U. (2015). Carbon Nanotubes and Carbon Nanofibers Fabricated on Tubular Porous Al_2_O_3_ Substrates. Carbon.

[11] Rönsch S., Schneider J., Matthischke S., Schlüter M., Götz M., Lefebvre J., Prabhakaran P., Bajohr S. (2016). Review on Methanation—From Fundamentals to Current Projects. Fuel.

[12] Tian C., Huang A. (2022). Synthesis of a Cu/Zn-BTC@LTA Derivatived Cu–ZnO@LTA Membrane Reactor for CO_2_ Hydrogenation. J. Membr. Sci..

[13] Rieck F., Mundstock A., Richter H., Huang A., Kißling P., Hindricks K.D.J., Behrens P., Caro J. (2022). Controlled Methylamine Synthesis in a Membrane Reactor Featuring a Highly Steam Selective K+-LTA Membrane. Microporous Mesoporous Mater..

[14] Krishna R. (1990). Multicomponent Surface Diffusion of Adsorbed Species: A Description Based on the Generalized Maxwell-Stefan Equations. Chem. Eng. Sci..

[15] Monsalve Bravo G.M. (2016). Engineering Models of Permeation in Mixed-Matrix Membranes. Ph.D. Thesis.

[16] Cardoso S.P., Azenha I.S., Portugal I., Lin Z., Rodrigues A.E., Silva C.M. (2017). Single and Binary Surface Diffusion Permeation through Zeolite Membranes Using New Maxwell-Stefan Factors for Dubinin-type Isotherms and Occupancy-Dependent Kinetics. Sep. Purif. Technol..

[17] Cavenati S., Grande C.A., Rodrigues A.E., Kiener C., Müller U. (2008). Metal Organic Framework Adsorbent for Biogas Upgrading. Ind. Eng. Chem. Res..

[18] Choi B.U., Choi D.K., Lee Y.W., Lee B.K., Kim S.H. (2003). Adsorption Equilibria of Methane, Ethane, Ethylene, Nitrogen, and Hydrogen onto Activated Carbon. J. Chem. Eng. Data.

[19] Hao S., Chu W., Jiang Q., Yu X. (2014). Methane Adsorption Characteristics on Coal Surface above Critical Temperature through Dubinin–Astakhov Model and Langmuir Model. Colloids Surf. A Physicochem. Eng. Asp..

[20] Ito M., Nishihara H., Yamamoto K., Itoi H., Tanaka H., Maki A., Miyahara M.T., Yang S.J., Park C.R., Kyotani T. (2013). Reversible Pore Size Control of Elastic Microporous Material by Mechanical Force. Chem.-Eur. J..

[21] Men’shchikov I.E., Shkolin A.V., Fomkin A.A., Khozina E.V. (2021). Thermodynamics of Methane Adsorption on Carbon Adsorbent Prepared from Mineral Coal. Adsorption.

[22] Marco-Lozar J.P., Kunowsky M., Suárez-García F., D. Carruthers J., Linares-Solano A. (2012). Activated Carbon Monoliths for Gas Storage at Room Temperature. Energy Environ. Sci..

[23] Rother J., Fieback T. (2013). Multicomponent Adsorption Measurements on Activated Carbon, Zeolite Molecular Sieve and Metal–Organic Framework. Adsorption.

[24] Takagi H., Hatori H., Soneda Y., Yoshizawa N., Yamada Y. (2004). Adsorptive Hydrogen Storage in Carbon and Porous Materials. Mater. Sci. Eng. B.

[25] Voskuilen T.G., Pourpoint T.L., Dailly A.M. (2012). Hydrogen Adsorption on Microporous Materials at Ambient Temperatures and Pressures up to 50 MPa. Adsorption.

[26] NIST/ARPA-E Database of Novel and Emerging Adsorbent Materials. https://adsorption.nist.gov/isodb/index.php#home.

[27] Kämnitz S., Simon A., Richter H., Weyd M., Lubenau U., Geisler T., Voigt I. (2022). Hydrogen Conditioning Using Nanoporous Inorganic Membranes. Chem. Ing. Tech..

[28] Burket C.L. (2007). Genesis and Evolution of Porosity and Microstructure in Nanoporous Carbon Derived from Polyfurfuryl Alcohol. Ph.D. Thesis.

[29] Gao X., Bonilla M.R., da Costa J.C.D., Bhatia S.K. (2012). The Transport of Gases in Macroporous *α*-Alumina Supports. J. Membr. Sci..

[30] Gao X., Bonilla M.R., da Costa J.C.D., Bhatia S.K. (2013). The Transport of Gases in a Mesoporous *γ*-Alumina Supported Membrane. J. Membr. Sci..

[31] Gregg S., Sing K. (1982). Adsorption, Surface Area and Porosity.

[32] Rouquerol J., Rouquerol F., Llewellyn P., Maurin G., Sing K. (2013). Adsorption by Powders and Porous Solids: Principles, Methodology and Applications.

[33] Del Moral P., Doucet A., Jasra A. (2006). Sequential Monte Carlo Samplers. J. R. Stat. Soc. Ser. B Stat. Methodol..

[34] Geris L., Gomez-Cabrero D. (2016). Uncertainty in Biology: A Computational Modeling Approach.

[35] Clarkson W. (2014). HOWTO Estimate Parameter-Errors by Monte-Carlo. http://www-personal.umd.umich.edu/~wiclarks/AstroLab/HOWTOs/NotebookStuff/MonteCarloHOWTO.html.

[36] Andersson J.A.E., Gillis J., Horn G., Rawlings J.B., Diehl M. (2019). CasADi: A Software Framework for Nonlinear Optimization and Optimal Control. Math. Program. Comput..

[37] Do D.D. (1998). Adsorption Analysis: Equilibria and Kinetics.

[38] Krishna R., van Baten J.M. (2008). Onsager Coefficients for Binary Mixture Diffusion in Nanopores. Chem. Eng. Sci..

[39] Nagy E. (2018). Basic Equations of Mass Transport Through a Membrane Layer.

[40] Fu S., Sanders E.S., Kulkarni S.S., Wenz G.B., Koros W.J. (2015). Temperature Dependence of Gas Transport and Sorption in Carbon Molecular Sieve Membranes Derived from Four 6FDA Based Polyimides: Entropic Selectivity Evaluation. Carbon.

[41] Gilliland E.R., Baddour R.F., Perkinson G.P., Sladek K.J. (1974). Diffusion on Surfaces. I. Effect of Concentration on the Diffusivity of Physically Adsorbed Gases. Ind. Eng. Chem. Fundamen..

[42] Builes S., Sandler S.I., Xiong R. (2013). Isosteric Heats of Gas and Liquid Adsorption. Langmuir.

[43] Tun H., Chen C.C. (2021). Isosteric Heat of Adsorption from Thermodynamic Langmuir Isotherm. Adsorption.

[44] Richter H., Voss H., Kaltenborn N., Kämnitz S., Wollbrink A., Feldhoff A., Caro J., Roitsch S., Voigt I. (2017). High-Flux Carbon Molecular Sieve Membranes for Gas Separation. Angew. Chem. Int. Ed..

