# Supplementary material for: Pure Hydrogen and Methane Permeation in Carbon-Based Nanoporous Membranes: Adsorption Isotherms and Permeation Experiments
Source: Membranes (Basel). 2024 May 26;14(6):123. doi: 10.3390/membranes14060123 (PMC11205412; doi:10.3390/membranes14060123)
Supplement: Supplementary file 1 [file membranes-14-00123-s001.zip › Monte Carlo Methodology.pdf]

Monte Carlo Methodology

Methodology of Monte Carlo simulation

1. **Parameter Estimation:** The parameters of the model were estimated using all available data points. The resulting parameter values were treated as the true values of the model parameters.
2. **Calculating Error:** The squared correlation coefficient (r2) was calculated as an estimate of the error in the parameter estimation. This value represents the proportion of the variance in the observed data that is explained by the model.
3. **Generating Trial Values:** Over 500 trials, new values of the dependent variable (y) were generated based on the estimated parameters and the error calculated in step 2. These new values were referred to as `y_Trial` values.
4. **Reestimating Parameters:** For each `y_Trial` value, the parameters of the model were reestimated. This process was repeated for all 500 trials.
5. **Calculating Uncertainty:** The 1 σ range of the results of the resulting curves was obtained. This range represented the region within which the true values of the dependent variable were expected to lie with a 68% confidence level.

The whole process is based on the tutorial in this webpage .
